# Cell Wall Glycans Mediate Recognition of the Dairy Bacterium Streptococcus thermophilus by Bacteriophages

**DOI:** 10.1128/AEM.01847-18

**Published:** 2018-11-15

**Authors:** Paula Szymczak, Sérgio Raposo Filipe, Gonçalo Covas, Finn Kvist Vogensen, Ana Rute Neves, Thomas Janzen

**Affiliations:** a Bacterial Physiology, R&D Microbial Platform, Chr. Hansen A/S, Hørsholm, Denmark; b Department of Food Science, University of Copenhagen, Frederiksberg, Denmark; c UCIBIO-REQUIMTE, Departamento de Ciências da Vida, Faculdade de Ciências e Tecnologia, Universidade Nova de Lisboa, Caparica, Portugal; d Laboratory of Bacterial Cell Surfaces and Pathogenesis, Instituto de Tecnologia Química e Biológica António Xavier, Universidade Nova de Lisboa, Oeiras, Portugal; The Pennsylvania State University

**Keywords:** Streptococcus thermophilus, bacteriophages, receptors, adsorption, cell wall, polysaccharides, glycans

## Abstract

Receptors on the cell surfaces of bacterial hosts are essential during the infection cycle of bacteriophages. To date, the phage receptors of the industrial relevant dairy starter bacterium Streptococcus thermophilus remain elusive. Thus, we set out to identify cell surface structures that are involved in host recognition by dairy streptococcal phages. Five industrial S. thermophilus strains sensitive to different phages (*pac* type, *cos* type, and the new type 987), were selected to generate spontaneous bacteriophage-insensitive mutants (BIMs). Of these, approximately 50% were deselected as clustered regularly interspaced short palindromic repeat (CRISPR) mutants, while the other pool was further characterized to identify receptor mutants. On the basis of genome sequencing data, phage resistance in putative receptor mutants was attributed to nucleotide changes in genes encoding glycan biosynthetic pathways. Superresolution structured illumination microscopy was used to visualize the interactions between S. thermophilus and its phages. The phages were either regularly distributed along the cells or located at division sites of the cells. The cell wall structures mediating the latter type of phage adherence were further analyzed via phenotypic and biochemical assays. Altogether, our data suggested that phage adsorption to S. thermophilus is mediated by glycans associated with the bacterial cell surface. Specifically, the *pac*-type phage CHPC951 adsorbed to polysaccharides anchored to peptidoglycan, while the 987-type phage CHPC926 recognized exocellular polysaccharides associated with the cell surface.

**IMPORTANCE** Streptococcus thermophilus is widely used in starter cultures for cheese and yoghurt production. During dairy fermentations, infections of bacteria with bacteriophages result in acidification failures and a lower quality of the final products. An understanding of the molecular factors involved in phage-host interactions, in particular, the phage receptors in dairy bacteria, is a crucial step for developing better strategies to prevent phage infections in dairy plants.

## Abstract (precis)

Streptococcus thermophilus is widely used in starter cultures for cheese and yoghurt production. During dairy fermentations, infections of bacteria with bacteriophages result in acidification failures and a lower quality of the final products. An understanding of the molecular factors involved in phage-host interactions, in particular, the phage receptors in dairy bacteria, is a crucial step for developing better strategies to prevent phage infections in dairy plants.

## INTRODUCTION

Streptococcus thermophilus, a thermophilic Gram-positive lactic acid bacterium (1), is one of the most common microorganisms used worldwide as a starter in the production of fermented foods, such as cheese and yoghurt (2, 3). The extensive use of S. thermophilus in dairy plants results in an increased probability of bacteriophage infections (4). Phage outbreaks represent the major cause of slow or faulty fermentations, frequently leading to a lower quality of dairy products (2, 5).

Diverse treatments have been applied to minimize phage infections in the dairy environment. Predominant approaches include chemical and physical methods for equipment sanitation (6) as well as culture replacement and strain rotation programs (7). The latter require strains with identical technological performance but different phage sensitivities (8). This requirement can be met by generating bacteriophage-insensitive mutants (BIMs). The methods to generate BIMs for commercial applications include a secondary culture method (8), serial passage in the presence of high phage titers (9), and chemical mutagenesis (10). Additionally, genetic engineering approaches (11, 12) can be used, in accordance with local legislation. In BIMs of S. thermophilus, the mechanisms commonly mediating resistance involve clustered regularly interspaced short palindromic repeat (CRISPR)-Cas systems or restriction-modification (R-M) systems (11, 13, 14), but abortive infection (Abi) (15) and prophage-carried superinfection exclusion (Sie) (16) have less commonly been identified.

CRISPR-Cas and R-M systems target specific genetic sequences of the invading phage (17). These mechanisms have limited robustness, because phages commonly acquire point mutations in their genomes to overcome bacterial immunity (4, 18–21). Additionally, phages acquire methylase genes (22) or produce proteins that inhibit CRISPR-Cas activity (23, 24). Therefore, BIMs in which the resistance is mediated by those mechanisms may not be suitable for industrial applications (3).

To obtain robust phage-resistant variants of S. thermophilus strains, strategies to select for BIMs with inhibited phage adherence to the bacterial cell walls were proposed (25). Resistance in these BIMs results from either modification or masking of the phage receptor structure (4, 26). Thus, an understanding of the interactions between the antireceptors of phages and their receptors present on the cell surface of an S. thermophilus bacterial strain is a determining factor for the development of phage-resistant cultures.

A knowledge of the structure and properties of bacterial cell walls is advantageous when studying the components recognized by phages. The cell walls of Gram-positive bacteria consist of a peptidoglycan (PG) layer that surrounds the cytoplasmic membrane and is decorated with other glycans and proteins (27). The cell wall glycans comprise two groups of cell surface-associated polysaccharides: (i) exocellular polysaccharides synthetized by the Wzy pathway encoded in the *eps* cluster (free exopolysaccharide [EPS] and capsular polysaccharide [CPS]) (28), and (ii) polysaccharides intercalated with PG (WPS; e.g., pellicles [29] or rhamnose-containing cell wall polysaccharides [30]). A third group of glycans are teichoic acids, classified into wall teichoic acids (WTA), which are covalently bound to PG (31), and lipoteichoic acids (LTA), which are anchored to the membrane (32).

In lactic acid bacteria, the cell wall components involved in the interactions between bacteria and their phages are best studied in Lactococcus lactis (3). A correlation between the receptor type present on the cell surface and the tail-tip morphology of the phage has been established (33). Members of two dominating groups of L. lactis phages, 936 and P335, which hold complex baseplate structures, recognize specific oligosaccharides of the highly variable pellicle (34–39). L. lactis phages from the c2 group have small tail tips (40) and use proteins, either PIP or YjaE, to act as receptors for the irreversible interaction with their hosts (7, 41). In Lactobacillus delbrueckii, LTA were designated the receptors for the phage LL-H (42, 43), interacting with a fiber located at the end of the phage tail (44).

To date, four types of S. thermophilus phages have been characterized (45–47). The two dominating types, the *cos*- and *pac*-type phages, are genetically distinct but display similar morphological characteristics, possessing long tails (more than 200 nm) with or without fibers on the tail tips (45, 46, 48). Phages belonging to the 5093 type have tails of similar lengths but terminate with globular baseplates (9, 45). The new 987 type comprises phages with short tails (120 to 150 nm) and complex baseplate structures (45, 47).

Phage receptors of S. thermophilus may be polysaccharides, teichoic acids, or proteins. Previous studies indicated a role of carbohydrates in the adsorption of phages to S. thermophilus (2, 49). Furthermore, the presence of CPS was reported to influence phage sensitivity in S. thermophilus (10), while a loss of the ropy phenotype, i.e., the ability to produce exocellular polysaccharides, was associated with the acquisition of phage resistance in a non-CRISPR BIM (14). To date, the identity of phage receptors in S. thermophilus remains elusive.

The aim of this study was to identify the phage receptors of S. thermophilus. To that end, we generated BIMs of industrial S. thermophilus strains and identified mutations putatively involved in phage recognition. Phage-host interactions were visualized via superresolution structured illumination fluorescence microscopy. Biochemical assays were performed to identify the macromolecules recognized by phages with different antireceptor structures. This report establishes the identity of phage receptors at the bacterial cell surface of S. thermophilus.

## RESULTS

### Industrial strains and phages selected to unveil phage receptors.

Five industrial S. thermophilus strains, representing different lytic groups, and their phages were selected for the study (Table 1). These strains are components of commercial dairy starter cultures and are sensitive to phages that have different antireceptor structures on their tail tips (see Fig. S1 in the supplemental material). Strain STCH_09 has a texturizing phenotype (free EPS producer) and is infected by phage CHPC1057 (*pac* type), which terminates with a tail fiber. Strains STCH_12, STCH_13, STCH_14, and STCH_15 are fast-acidifying nontexturizing bacteria that are implemented in processes of cheese fermentation. They are sensitive to phages CHPC951 (*pac* type), CHPC1014 (*cos* type), CHPC1046 (*cos* type), and CHPC926 (987 type), respectively. The first three phages have tail fibers, while the tail of CHPC926 ends with a complex baseplate. The diverse characteristics of these strains and their phages were expected to bring a broad perspective on cell wall structures recognized by S. thermophilus phages.

**TABLE 1:** List of S. thermophilus strains and phages from the Chr. Hansen A/S collection used in this study

Strain	Characteristic	Dairy production	Sensitive toward phage:	Phage type
STCH_09	Fast-acidifying strain with a texturizing phenotype	Fermented milks	CHPC1057	*pac*
STCH_12	Fast-acidifying strain	Cheese	CHPC951	*pac*
STCH_13	Fast-acidifying strain	Cheese	CHPC1014	*cos*
STCH_14	Fast-acidifying strain	Cheese	CHPC1046	*cos*
STCH_15	Fast-acidifying strain	Cheese	CHPC926	987

### Putative receptor mutants have mutations in genes encoding glycan biosynthetic pathways.

To identify mutations in genes potentially encoding phage receptors, a range of spontaneous bacteriophage-insensitive mutants (BIMs) was generated from the wild-type (WT) strains by challenging them with their respective phages (Table 1). BIMs of STCH_09, STCH_12, STCH_13, and STCH_14 were successfully obtained using a plating method, while BIMs of STCH_15 were generated by growing in liquid medium (see Materials and Methods). The efficiency of BIM formation differed between strains (Table 2).

**TABLE 2:** Number of BIMs from five backgrounds generated and selected in this study

Strain	No. of generated BIMs^*a*^	No. of CRISPR BIMs	No. of sequenced non-CRISPR BIMs
STCH_09	39	13	7
STCH_12	16	6	10
STCH_13	47	31	4
STCH_14	16	10	3
STCH_15	24	7	7

a BIMs, bacteriophage-insensitive mutants.

In this study, we selected for BIMs harboring receptor defects; thus, isolates that became phage resistant due to the activation of a CRISPR-Cas system were excluded. To that end, a colony PCR was performed with primers specific for the CRISPR1, CRISPR2, and CRISPR3 loci in S. thermophilus (50). BIMs with spacer acquisitions were visualized on an agarose gel as a product of a larger size compared to that from the WT (data not shown). In total, 67 of 142 tested BIMs were rejected from the investigation as potential CRISPR mutants (Table 2).

**FIG 1:**
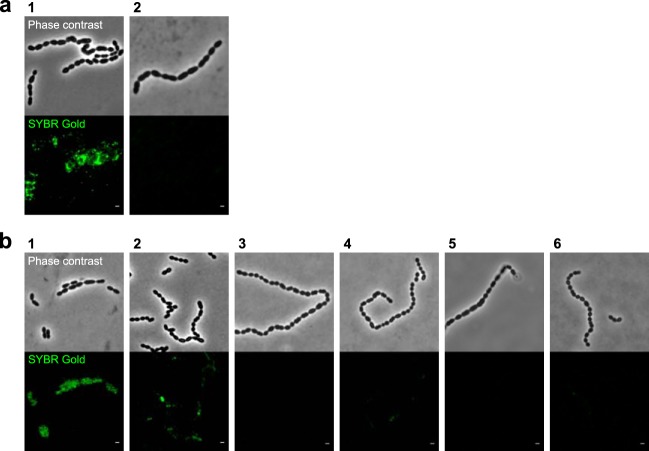
Changes in phage binding to S. thermophilus wild-type strains and their phage-resistant mutants. Phage DNA was labeled with SYBR Gold, and the green fluorescence was visualized. (a) Phage CHPC1057 adsorbs to its host STCH_09 (panel 1) and does not adsorb to STCH_09_BIM (panel 2). (b) Phage CHPC926 adsorbs to its host STCH_15 (panel 1), has reduced adsorption with STCH_15_BIM_2 (panel 2), and does not adsorb to STCH_15_BIM_1, STCH_15_BIM_3, STCH_15_BIM_4, or STCH_15_BIM_5 (panels 3 to 6, respectively). Scale bars, 1 μm.

The remaining BIMs were subjected to phenotypic assays to select candidates with effective phage resistance and properties of receptor mutants. A spot test was used to assess the reduction of phage titers in non-CRISPR BIMs. The mutants selected for sequencing did not form plaques with their phages, confirming the activation of phage resistance mechanisms independent of CRISPR-Cas systems. An impairment of phage adhesion to non-CRISPR BIMs was examined by mixing BIMs with SYBR Gold-labeled phages and screening them under a fluorescence microscope. BIMs of strains STCH_09 and STCH_15 had reduced phage adsorption compared to that of the WTs (Fig. 1). Mutants of strains STCH_14 and STCH_15 formed elongated chains compared to those of the WTs, as shown in Fig. S2. These changes in chain lengths are associated with alterations of cell surface properties (27, 30, 31). Changes in the production of free EPS by STCH_09 and its derivatives were examined with a volumetric pipette viscosity test. Four of seven BIMs of STCH_09 had altered viscosity compared to that of the WT (see Table S1). On the basis of this characterization, we selected 31 non-CRISPR mutants from the five backgrounds displaying different phenotypic traits (Table 2).

Mutations in genes encoding glycan biosynthetic pathways were detected in the genomes of selected BIMs. The gene modifications are described in Table 3 and Table S2. Briefly, nucleotide substitutions that lead to amino acid substitutions in two glycosyltransferases were detected in STCH_09_BIM. The glycosyltransferase *epsH* belongs to the exocellular polysaccharide (*eps*) operon (28), while the other glycosyltransferase belongs to the rhamnose-containing polysaccharide (*rgp*) operon (30). Mutations in genes of *eps* operons were also found in BIMs of fast-acidifying strains STCH_13, STCH_14, and STCH_15. An insertion of three nucleotides in the *epsD* gene of STCH_13_BIM led to the introduction of an additional amino acid in the gene product, while a nucleotide substitution in the *epsE* gene of STCH_14_BIM led to gene truncation. A complete deletion of the priming glycosyltransferase *epsE* gene was observed in STCH_15_BIM_1. The four other mutants of STCH_15 had unique mutations in the glycosyltransferase *epsK* gene, which resulted either in a amino acid substitution, as for STCH_15_BIM_2, or in frameshift and nonsense mutations and ultimately nonfunctional proteins, as for STCH_15_BIM_3, STCH_15_BIM_4, and STCH_15_BIM_5. The BIMs of STCH_15 had either reduced or lacked phage adsorption as observed under a fluorescence microscope (Fig. 1b). In conclusion, the detected mutations had the potential to affect the production of cell surface-associated polysaccharides, resulting in a loss of phage adherence to some BIMs or, for the BIMs with unchanged phage adsorption, possibly affecting other parameters such as phage DNA injection (14).

**TABLE 3:** List of mutations in genes encoding glycan biosynthesis pathways detected in BIMs generated in this study

BIM^*a*^	Mutated gene	Putative gene function	Type of mutation	AA^*b*^ position	Phenotypic change compared to WT
STCH_09_BIM	*epsH*	Glycosyltransferase	AA substitution	262	Reduced adsorption, enhanced viscosity
	*rgp*	Glycosyltransferase	Gene truncation	197	
STCH_13_BIM	*epsD*	Tyrosine-kinase	AA insertion	160	Not detected
STCH_14_BIM	*epsE*	Priming glycosyltransferase	Gene truncation	37	Long chains
STCH_15_BIM_1	*epsE*	Priming glycosyltransferase	Gene deletion	Whole gene	Reduced adsorption, long chains
STCH_15_BIM_2	*epsK*	Glycosyltransferase	AA substitution	75	Reduced adsorption, long chains
STCH_15_BIM_3			Gene truncation	93	Reduced adsorption, long chains
STCH_15_BIM_4			Gene truncation	82	Reduced adsorption, long chains
STCH_15_BIM_5			Gene truncation	93	Reduced adsorption, long chains

a BIM, bacteriophage-insensitive mutant.

b AA, amino acid.

### Phage receptors are either located at the septum or distributed uniformly around the cell surface.

With the assumption that cell wall glycans are involved in phage adhesion to S. thermophilus, we questioned if phage receptors were located in specific areas on the cell surface and if the location was correlated with the phage antireceptor. Information on phage binding spots might assist in identifying structures recognized by phages, since diverse cell wall structures in Gram-positive bacteria are differently distributed around the cells. For instance, the proteins involved in cell division are located at the septum (51); WTA may locate at the septum or distribute irregularly in mature PG (52–54), while cell surface-associated polysaccharides evenly surround the cells (29, 55). To specify the receptor locations on the cell surface, phage-host interactions were visualized via conventional and superresolution fluorescence microscopy.

**FIG 2:**
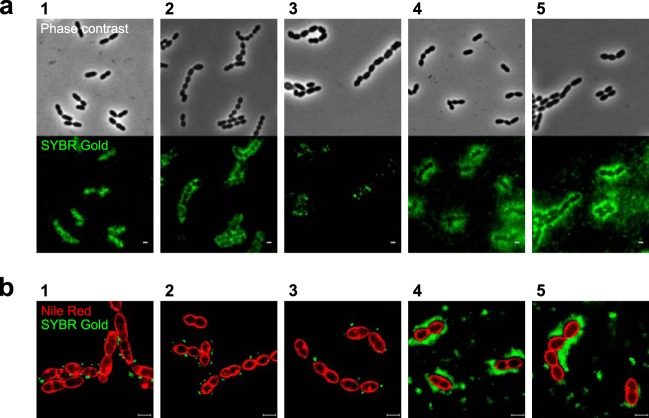
Fluorescence imaging of phage binding to S. thermophilus strains. (a) Adsorption of phages to their hosts was visualized with a conventional fluorescence microscope after labeling phage DNA with SYBR Gold. (b) Superresolution structured illumination microscopy (SR-SIM) images of bacterial cells stained with Nile red (red) and mixed with SYBR Gold DNA-labeled phages (green). Panels with phages and their host strains: 1, CHPC926 and STCH_15; 2, CHPC951 and STCH_12; 3, CHPC1057 and STCH_09; 4, CHPC1014 and STCH_13; 5, CHPC1046 and STCH_14. Two binding patterns are observed: spotty (panel numbers 1, 2, and 3) or diffused (panel numbers 4 and 5). Scale bars, 1 μm.

**FIG 3:**
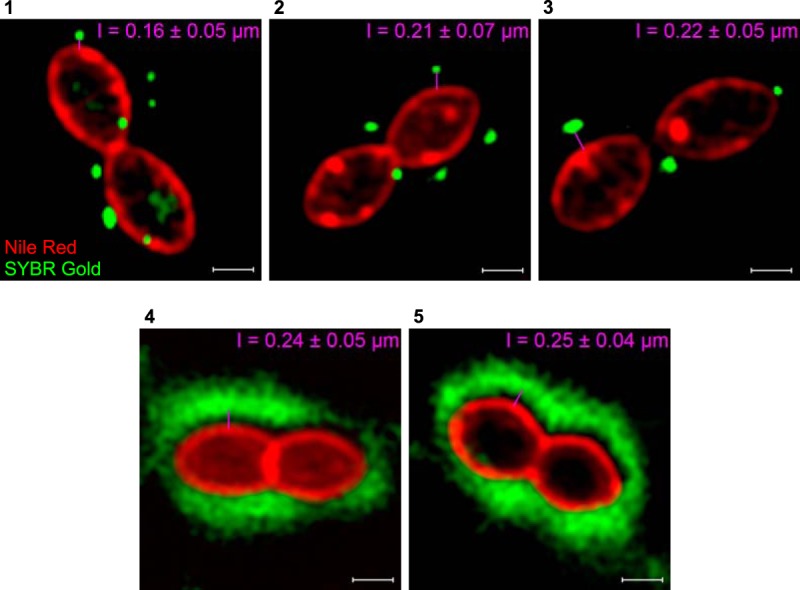
Superresolution structured illumination microscopy (SR-SIM) images of phage binding to S. thermophilus strains. The pink lines indicate the distance between phage capsids, containing SYBR Gold-labeled DNA (green), and bacterial membranes, stained with Nile red (red). The presented values correspond to the lengths of phage tails and are the averages from 80 measurements. Panels with phages and their host strains: 1, CHPC926 and STCH_15; 2, CHPC951 and STCH_12; 3, CHPC1057 and STCH_09; 4, CHPC1014 and STCH_13; 5, CHPC1046 and STCH_14. Scale bars, 0.5 μm.

Two distinct patterns of phage adhesion to the host cells were observed via microscopy techniques. Phage particles, for which their DNA was labeled with SYBR Gold, were either irregularly distributed throughout the cell surface or created a dispersed halo that surrounded the host cells when visualized with a conventional fluorescence microscope (Fig. 2a). The spotty and diffused binding patterns were additionally verified by using superresolution structured illumination microscopy (SR-SIM) (Fig. 2b and S3). The fluorescent signals of phages CHPC926, CHPC951, and CHPC1057 were localized at division sites of the host cells: at the septum, in the areas where a septal membrane ring began to build, or where the cell wall has been produced and split (Fig. 2b, panels 1 to 3). *cos*-type phages CHPC1014 and CHPC1046, which hold fibers at the ends of their tails, displayed dispersed fluorescent signals distributed uniformly along the cells (Fig. 2b, panels 4 and 5). The type of binding did not depend on the ratio of phages toward their hosts. The test was performed at multiplicities of infection (MOI) of approximately 10 and 1, and differences between the two conditions were not observed; i.e., the nature of adsorption, spotty or diffused, remained unaffected (data not shown). Furthermore, the distance between a phage capsid, which contained DNA labeled with a green fluorophore (SYBR Gold), and a bacterial membrane stained with a red fluorophore (Nile red) is in accordance with the values determined by electron microscopy for the length of the phage tails and provided additional evidence that the green fluorescence signal represents phage binding (Fig. 3).

### Binding of a phage at the septum is mediated by cell wall glycans.

We wanted to identify which cell wall components were involved in the phage adsorption at the septal areas of the cells, because we commonly observed this type of phage-host interaction in S. thermophilus and we detected spotty adsorption with phages having different tail-tip morphologies. Thus, two strains with phage receptors located at the septa were selected for further study: STCH_12, infected by *pac*-type phage CHPC951 with a tail fiber, and STCH_15, infected by 987-type phage CHPC926 with a baseplate. Additionally, to verify the effects of mutations of *eps* genes on phage receptors, STCH_15_BIM_1 and STCH_15_BIM_2 were used in the assays.

To assess the involvement of different cell wall components in phage recognition, the selected S. thermophilus strains were treated as described in Materials and Methods (see Table S3) to deplete sequentially cellular components: (i) surface enzymes, membrane, and membrane proteins (purification step no. 2), (ii) LTA and cell wall proteins (purification step no. 3), (iii) WTA, WPS, and CPS (purification step no. 4). We assumed that exocellular polysaccharides loosely associated with the cell surface detached gradually in the multiple centrifuging steps applied in the preparation of cellular fractions. Subsequently, phage binding to the isolated cellular fractions was verified under a fluorescence microscope.

**FIG 4:**
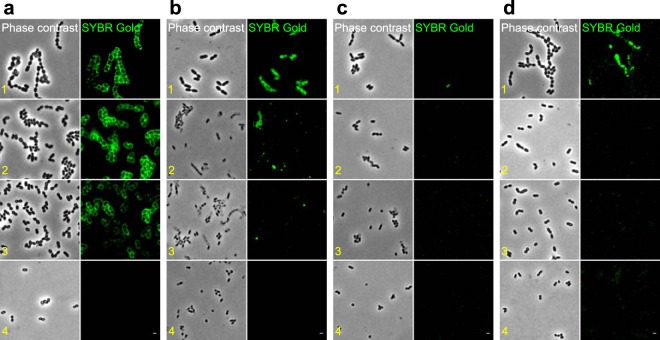
Fluorescence imaging of phage binding to cellular fractions of S. thermophilus. Phage DNA was labeled with SYBR Gold and the green fluorescence was visualized. (a) Phage CHPC951 was mixed with cellular fractions of STCH_12. Phage CHPC926 was mixed with cellular fractions of STCH_15 (b), cellular fractions of STCH_15_BIM_1 (c), or cellular fractions of STCH_15_BIM_2 (d). The following samples were used: 1, cells in exponential phase; 2, cells devoid of surface enzymes, membranes, and membrane proteins; 3, purified cell walls; 4, purified peptidoglycan. Scale bars, 1 μm.

Phages with different antireceptor structures attached to different cellular fractions of their hosts. Phage CHPC951 adsorbed to the surface of strain STCH_12 until purification step no. 4 was applied (Fig. 4a, panel 4). The adsorption of phage CHPC926 to the surface of strain STCH_15 was gradually reduced after purification steps no. 2 and 3 (Fig. 4b, panels 2 and 3, respectively). Thus, phage CHPC951 with a tail fiber established a binding complex with one of the cell wall glycans but not with PG. Phage CHPC926 with a baseplate interacted either with cell wall proteins, LTA, or exocellular polysaccharides associated with the cell surface.

Exocellular polysaccharides were the most probable phage receptors of STCH_15, since its BIMs held mutations in genes belonging to the *eps* operon. However, the production of extracellular matrix masking phage receptors might be another consequence of mutations of *eps* genes. Phage CHPC926 did not adsorb to any of the cellular fractions of STCH_15_BIM_1 (Fig. 4c), which indicated a substantial modification or even a deletion of a phage receptor. For STCH_15_BIM_2, phage adsorption was observed only with the cells in exponential phase (Fig. 4d, panel 1), which suggested that the phage bound to modified exocellular polysaccharides until these were depleted in subsequent purification steps.

**FIG 5:**
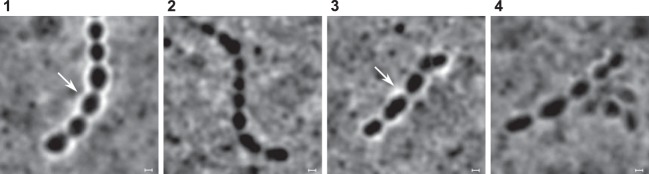
Microscopy images of India ink negatively stained S. thermophilus strains. Staining was performed for two wild-type strains and two phage-resistant mutants: 1, STCH_15; 2, STCH_15_BIM_1; 3, STCH_15_BIM_2; 4, STCH_12. Presence of capsular polysaccharide is indicated with arrows. Scale bars, 1 μm.

The effect of *eps* genes mutations on CPS formation was further assessed by the India ink negative staining method (Fig. 5). STCH_15 possessed CPS, visible as a transparent halo surrounding the cells (Fig. 5, panel 1). STCH_15_BIM_1, which was proposed to have phage receptor deletion, completely lacked CPS, because a transparent halo engulfing the cells was not detected (Fig. 5, panel 2). STCH_15_BIM_2 produced less CPS, as indicated by the less clear halo (Fig. 5, panel 3). On the basis of the observations made for the two BIMs, the mutations in genes belonging to the *eps* operon in STCH_15 caused modifications in exocellular polysaccharides which act as the phage receptor for the CHPC926 phage with a baseplate.

Macromolecules such as WTA, WPS, or CPS might mediate the adsorption of phage CHPC951 with a tail fiber. Thus, we evaluated the host potential to synthetize these molecules by searching for genes encoding their biosynthetic pathways in the genome. All S. thermophilus strains possessed two polysaccharide gene clusters: the *eps* operon (28) and the *rgp* operon (30). However, analogs of the *tag* and *tar* genes essential for WTA synthesis were missing; thus, WTA were excluded as receptors (31). In addition, STCH_12 showed no halo in response in the India ink staining test, indicating very low production or absence of CPS (Fig. 5, panel 4). Thus, it is reasonable to propose that the putative receptors for fiber-ending phage CHPC951 are polysaccharides intercalated with PG, most likely WPS encoded by the *rgp* operon.

The PG was excluded as a phage receptor in S. thermophilus, since none of the phages were found to bind the purified PG (Fig. 4a to d, no. 4 panels). The PG compositions of S. thermophilus progenitors and mutants were identical, as determined by the high-pressure liquid chromatography (HPLC) profiles due to the amount and type of muropeptides released with PG that was incubated with PG hydrolase (see Fig. S4). Moreover, the monosaccharides present in the glycan chain, *N*-acetylglucosamine and *N*-acetylmuramic acid, were identified in the PG at the same relative concentrations for the WTs and BIMs (see Fig. S5a). Thus, PG did not seem to be the structure recognized by S. thermophilus phages.

### Composition of saccharides in glycan chains is crucial for phage adsorption.

**FIG 6:**
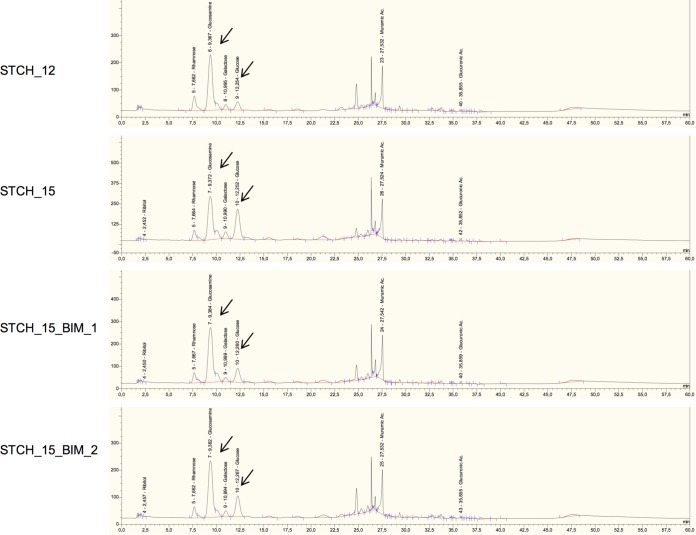
Profiles of monosaccharide compositions in purified cell walls isolated from S. thermophilus strains. HPAEC-PAD analyses were performed for cell walls purified from two wild-type strains (STCH_12 and STCH_15) and two phage-resistant mutants (STCH_15_BIM_1 and STCH_15_BIM_2). Standards for different saccharides were eluted under the same conditions to enable peak identification. Differences in the heights of glucosamine and glucose peaks are indicated with arrows.

Cell wall glycans commonly differ in linkage, branching, and substitutions (28, 30, 56). We questioned if the specific composition of saccharides in chains was necessary for the efficient binding of the phages. To that end, the monosaccharide compositions were analyzed in samples collected during the purification of cellular fractions of WTs and BIMs (Table S3). Although the cell walls of WTs and BIMs were composed of the same monosaccharide types, the ratios of carbohydrates varied between the strains. The cellular fractions of S. thermophilus strains were composed of monosaccharides typical for the PG structure, *N*-acetylglucosamine and *N*-acetylmuramic acid, as well as rhamnose, galactose, and glucose. However, STCH_12 had the largest amount of glucosamine and the smallest amount of glucose among all the strains, while STCH_15 had less glucosamine and more glucose than its BIMs (Fig. 6; Fig. S5b). The calculation was based on the profiles generated for purified cell walls with polysaccharides anchored to PG (WPS and CPS). The differences in monosaccharide profiles of the cell walls indicated that WTs and BIMs had unique glycan compositions that were linked to the differences in phage sensitivities.

## DISCUSSION

In this work, we obtained evidence supporting the involvement of cell wall glycans on phage adsorption to dairy strains of S. thermophilus. The macromolecular receptors are either regularly distributed along the cells (dispersed pattern) or located at septal areas of the cells (spotty pattern). The spotty pattern of adherence is mediated by different cell wall components: the fiber-ending phage CHPC951 adsorbs to polysaccharides anchored to PG, while the baseplate phage CHPC926 binds to exocellular polysaccharides associated with the cell surface. On the basis of the results from a combination of genetic and biochemical approaches, we propose that Wzy-dependent exocellular polysaccharides and WPS are phage receptors in S. thermophilus.

In BIMs of S. thermophilus, the recurrence of mutations in genes involved in glycan biosynthetic pathways, e.g., those encoding glycosyltransferases, the priming glycosyltransferase, and the bacterial tyrosine-kinase, strongly indicates a role of cell surface glycans in the phage-host interaction. This view is in line with an observed loss of phage adsorption to several of these BIMs. Previously, free EPS contributing to a ropy phenotype was reported to be related to phage sensitivity in S. thermophilus (14). The results of our study show that S. thermophilus acquires mutations in genes of the *eps* operon (biosynthesis of free EPS and CPS) as a response to phage infections, independent of a texturizing phenotype. Moreover, mutations in glycan biosynthetic pathways genes occurred in response to phages of different types, including the dominating *pac*- and *cos*-type phages with tail fibers as well as 987-type phages with baseplate at the tail tips. With a consideration of these findings, one may speculate that in S. thermophilus, cell wall glycans are universal receptors for phages.

Approximately 50% of the BIMs generated by challenging S. thermophilus with their phages were CRISPR mutants, a result that was not surprising considering that the activation of intracellular defense strategies is the predominant response to phage attack (3, 14). Mutations in R-M systems were rare in the fraction of non-CRISPR BIMs. Of these, circa 25% carried mutations in glycan biosynthesis genes. The impact of these mutations on the performance of the strains needs to be evaluated prior to industrial use. Moreover, to link these mutations with the reduced phage sensitivity and loss of adsorption, the inactivation of the target gene in the mother strain or the restoration of the phenotype by the introduction in *trans* of the wild-type gene can be envisioned. Although these genetic approaches commonly applied to domesticated strains might be appealing, they are difficult to implement in industrial strains that are not genetically amenable and for which tools for genetic manipulation are largely unavailable (57). We assumed that the complementation of gene mutations in the studied strains would be challenging. Therefore, we resorted to phenotypic and biochemical approaches to further substantiate our hypothesis that cell wall glycans act as phage receptors in S. thermophilus.

The common S. thermophilus *pac*- and *cos*-type phages with tail fibers (e.g., CHPC951) likely bind to polysaccharides anchored to cell walls. This observation conflicts with the previous idea that phages with simple antireceptors bind to conserved structures with repetitive units, such as proteins or teichoic acids (3, 33). The S. thermophilus strains used in this study contain in their genome sequences two operons involved in glycan biosynthesis pathways, namely, the *eps* operon encoding the biosynthetic pathway for the synthesis of free EPS and/or CPS and the *rgp* operon for the synthesis of WPS intercalated with PG. The absence of genes required for the synthesis of WTA is in agreement with the view that S. thermophilus does not synthetize WTA (30, 58), ruling out this macromolecule as a possible phage receptor. Furthermore, adsorption assays with purified cellular fractions enabled the exclusion of proteins, LTA, and purified PG as phage receptors for the strain infected by the fiber-ending phage. In this line of reasoning, we singled out polysaccharides anchored to PG as the only surface glycans that could interact with phage CHPC951.

Our results suggest that phages with baseplates (e.g., CHPC926) recognize CPS on the cell surface. Indeed, the spatial arrangement of baseplates is required to bind complex structures of heteropolysaccharides, such as those produced by the Wzy pathway (free EPS and CPS) or WPS (3, 33). In line with this, cell wall-associated polysaccharides in L. lactis are recognized by phages from the group P335 (34, 36, 38, 59), which are genetically related to 987-type S. thermophilus phage CHPC926 (45) Although proteins could be a secondary factor that mediates efficient binding, as in the case of L. lactis phages from the group c2 (7, 41), mutations in genes coding for putative membrane proteins were not found in sequenced BIMs, and thus proteins are unlikely required for phage-host interactions in S. thermophilus. Mutations in *eps* genes resulted in the production of an altered polysaccharide in the BIMs, as denoted by the monosaccharide composition, which correlated with the absence or reduction of phage adherence to the cellular fractions of the BIMs. As for the WT, phage binding was not detected in purified cell walls with intercalated WPS. Altogether, exocellular polysaccharides associated with cell walls (most likely CPS) are the structures mediating the adsorption of phage CHPC926 with a baseplate.

The type of adsorption, spotty or dispersed, and the phage tail-tip morphology were not correlated. Phage adsorption at the septal areas of the cells (spotty pattern) was observed for the phage with a baseplate as well as *pac*-type phages with tail fibers, while dispersed adsorption was observed for two *cos*-type phages with tail fibers. The two adsorption patterns might indicate that different types of S. thermophilus phages recognize distinct cell wall structures, that the same macromolecule displays different conformation/folding depending on location, or that the locations of the recognized macromolecules on the cell surface differ between S. thermophilus strains. The latter alternative seems more plausible, as substantiated below. S. thermophilus strain STCH_12 is sensitive to a number of *cos*- and *pac*-type phages with fiber-ending tails. Interestingly, a spotty pattern of adsorption to this strain was observed with all infective phages, independently of their type (data not shown). Thus, the macromolecule/structure recognized by phages of STCH_12 was located at the septum, supporting the view that the location of the receptor macromolecule is strain dependent. In summary, as with phages with a baseplate, fiber-ending phages also recognize cell surface glycans, namely, exocellular polysaccharides.

On the basis of our fluorescence microscopy results on adsorption patterns, polysaccharides can be either located at the septum or distributed evenly on the cell surface. The underlying mechanisms behind this phenomenon need to be investigated. However, the differential location of cell wall glycans at the surfaces of Gram-positive bacteria has been documented. In Bacillus subtilis, WTA are only detected at the septum (52), while in Staphylococcus aureus, they either are associated with the mature PG (54) or have two forms, one located at the septum and another distributed throughout the cell (53).

However, with the knowledge that antireceptor structures of *cos*- and *pac*-type phages are different, we cannot exclude that the two adsorption patterns indicate that different receptors are recognized. Fibers at the end of the tail are expected to mediate the primary interaction with macromolecules on the cell surface, while more distant tail-tip structures might be necessary for the efficient binding and phage DNA injection, similar to the model of interaction proposed for Lactobacillus delbrueckii and its phage LL-H (44). Since morphological differences might not be visible under the electron microscope, more information on the phage structures involved in host recognition is necessary for understanding the difference in adsorption patterns of phages with outwardly identical tail fibers.

In this study, SR-SIM was used to track phage-host interactions. This novel application is advantageous over standard methods for testing phage adsorption, such as conventional fluorescence microscopy, because the high resolution enables a more detailed view of phage binding spots. The combination of superresolution microscopy techniques with the understanding of the complex nature of the cell wall dynamics and structures of phage antireceptors is expected to add value for exploring phage-host interactions.

In conclusion, the results of this study provide evidence that exocellular polysaccharides and WPS are involved in phage adsorption in S. thermophilus. The identification of phage receptors of S. thermophilus will contribute to future strategies aimed to design robust phage-resistant dairy starter cultures.

## MATERIALS AND METHODS

### Bacteria, phages, and growth conditions.

The Streptococcus thermophilus strains and phages used for this study are listed in Table 1. The strains were stored at −40°C in growth medium supplemented with 15% (wt/vol) glycerol and cultured overnight at 37°C in LM17 broth (M17 broth [Oxoid, Denmark] with 2% [wt/vol] lactose) or anaerobically at 37°C on LM17 agar plates (M17 agar [Oxoid] with 2% [wt/vol] lactose). For the bacterial cells used for tests with phages, the growth medium was additionally supplemented with 10 mM CaCl_2_ and 10 mM MgCl_2_ (LM17-Ca/Mg). Streptococcus pneumoniae strain Pen6 (60), which was used as a control for the procedure of subtracting cellular components, was stored at −80°C in growth medium supplemented with 15% (wt/vol) glycerol and cultured in a casein-based semisynthetic medium (C+Y) at 37°C as described previously (61).

The phages were propagated as previously described (45) and stored at 4°C. Phage titers as well as the host ranges of investigated phages with bacterial strains were determined with the double agar overlay spot test, as described previously (62). After an overnight incubation under the appropriate growth conditions, the PFU per milliliter was calculated.

Bacteriophage-insensitive mutants (BIMs) were formed via two methods. The plating method was adapted from a published protocol (9), where an overnight culture of a sensitive host was mixed with adequate phages at a multiplicity of infection (MOI) of ≥1 (ratio of PFU to CFU per milliliter), plated in soft top agar (LM17-Ca/Mg broth and agar mixed 1:1), and monitored for the appearance of phage-resistant colonies after 24 to 48 h of incubation under the growth conditions. If no colonies grew, the MOI was reduced by mixing the bacteria with a diluted phage lysate, and the procedure was repeated. To increase the probability that the generated BIMs would acquire unique mutations, several single colonies of each wild-type (WT) strain were inoculated in individual tubes and plated on separate plates after mixing with adequate phages. Due to the inefficient lysis with one of the phages used in the study, the secondary culture method was performed (8), where LM17-Ca/Mg broth was inoculated with a 1% overnight culture of a sensitive host, followed by the addition of adequate phages at an MOI of 10 or an MOI of 0.01 and incubating at 37°C. Surviving cells were collected at two time points, after 5 h and after 72 h of incubation, centrifuged at 15,000 × *g* for 10 min, resuspended in saline, mixed with adequate phages at an MOI of ≥1, plated in soft top agar, and monitored for the appearance of phage-resistant colonies after an overnight incubation under the growth conditions. BIMs generated in both assays were purified by streaking on LM17 agar plates and incubating under the growth conditions in three sequential repetitions.

### Volumetric pipette viscosity test.

To test for differences in the production of exocellular polysaccharides (free EPS) between the strain with texturizing phenotype and its BIMs, 250 ml of boiled milk was inoculated with 1% overnight cultures and incubated overnight at 37°C. The samples were cooled to room temperature, gently mixed, and pipetted with a 25-ml pipette. The time for an unforced flow through the pipette was measured in three repetitions. The thresholds for viscosity changes were as follows: 25 to 34 s (reduced viscosity), 35 to 44 s (normal viscosity), and 45 to 54 s (increased viscosity).

### Microscopy techniques.

Microscopy screening of overnight cultures was performed to detect changes in cell chain lengths between WTs and BIMs. The cells were immobilized on a thin layer of 1% agarose in PreC medium (63). Photographs were taken using a Zeiss Axioplan 2 microscope equipped with a Plan-Neofluar objective (100×, 1.3 oil, Ph3) and a Zeiss Axiocam 503 monocamera (Zeiss, Germany).

The presence of CPS was tested via the India ink negative staining technique (55) with the modification that a mix of 7 μl of India ink with 7 μl of fresh milk and 3 μl of bacterial sample was prepared on a microscopy slide. After air-drying, the samples were visualized under the Zeiss Axioplan 2 microscope, with the specifications described above. After acquisition, the photos were processed with ZEN software (black edition, version 14.0.0.201).

Changes in phage adsorption to the bacterial cell walls before and after the depletion of cellular components were visualized under a fluorescence microscope. Freshly propagated phages were mixed 1,000:1 (vol/vol) with a 10-fold-diluted SYBR Gold stock solution (Invitrogen, USA) and incubated overnight in the dark at 4°C (45, 64). Bacterial cultures at exponential phase (optical density at 600 nm [OD_600_] of 0.5) and samples obtained during the purification of cellular fractions in steps no. 1 to 4 (see Table S3 in the supplemental material) were mixed with stained phages at MOIs of approximately 10 and 1 in LM17-Ca/Mg. The mixtures were immobilized on a thin layer of 1% agarose in PreC medium as described above. Photographs were taken with a 1-s exposure by using a Zeiss Axio Observer microscope with a Plan-Apochromat objective (100×, 1.4 oil, Ph3). Images were acquired with a Retiga R1 charge-coupled-device (CCD) camera (QImaging, Canada) and Metamorph 7.5 software (Molecular Devices, USA). After acquisition, the images were processed with ImageJ software (65).

Phage binding patterns were visualized by superresolution structured illumination microscopy (SR-SIM) because of its improved resolution compared to that of conventional microscopy (66). Bacterial cultures at exponential phase (OD_600_ of 0.5) were stained with 2 μl/ml Nile red (Invitrogen), incubated for 5 min at room temperature with agitation in the dark, and washed twice with LM17-Ca/Mg. Membrane-stained bacterial cells were mixed with SYBR Gold-labeled phages (MOI ≥ 10) and mounted on a 1% PreC agarose pad as specified above. Imaging was performed on an Elyra PS.1 microscope (Zeiss) with a 561-nm laser at 50% power and a 50-ms exposure for Nile red and a 488-nm laser at 20% power and a 50-ms exposure for SYBR Gold. The images were acquired via five grid rotations, with a 34-mm grating period for the 561-nm laser and a 28-mm grating period for the 488-nm laser, followed by the reconstruction and processing with ZEN software (black edition, version 14.0.0.201).

Transmission electron micrograph images of phages were generated according to the method described previously (45).

### Depletion of cellular components.

The purification of cellular fractions was performed via the previously described method (67), with a modification that overnight cultures were subcultured into 2 liters of LM17 broth (for S. thermophilus) or 2 liters of C+Y medium (for S. pneumoniae) at an initial OD_600_ of 0.01 and grown until the OD_600_ was between 0.5 and 1.0 (step no. 1). Chemical and enzymatic treatments were applied to the bacterial cell walls to progressively remove different cell wall components. Briefly, the cells were boiled with sodium dodecyl sulfate (SDS) and then washed with Milli-Q water to obtain cells devoid of surface proteins and membranes (step no. 2). The sample was considered devoid of exocellular polysaccharides loosely associated with the cell surface, as they detached during the multiple centrifugations applied in this step. The subsequent treatment with enzymes, lithium chloride (LiCl), EDTA, and acetone was executed to remove components ionically bound to the cell wall, such as proteins, as well as LTA and intracellular components, i.e., DNA and RNA (step no. 3). The obtained cell walls with WTA, WPS, and CPS were treated with 46% hydrofluoric acid (HF) according to the previously described protocol (67) and incubated at 4°C for 72 h (step no. 4). In this step, cell surface glycans were separated from PG. Aliquots of the cellular fractions (Table S3) were stored at −20°C until further analyses were performed.

The monosaccharide compositions in samples collected during the purification of cellular fractions (Table S3) were examined by high-performance anion-exchange chromatography coupled with pulsed amperometric detection (HPAEC-PAD) as described previously (67). The volumes of samples used for hydrolysis with hydrochloric acid (HCl) prior to the injection in the column were 100 μl for cells collected at exponential phase, 40 μl for cells devoid of surface enzymes, membranes, and membrane proteins (this amount corresponded to approximately 1 × 10^9^ cell equivalents), 20 μl at a concentration of 40 mg/ml for purified cell walls, and 20 μl at a concentration of 20 mg/ml for purified PG. The standards for monosaccharides typically present in cell walls of Gram-positive bacteria (glucosamine, *N*-acetylglucosamine, muramic acid, *N*-acetylmuramic acid, rhamnose, glucose, galactose, ribitol, fucose, ribose, mannose, and glucuronic acid [28, 56]) were eluted under the same conditions to enable the identification of chromatogram peaks.

Muropeptides present in purified PG were prepared and analyzed by reverse phase HPLC as previously described (67).

### Molecular techniques.

To exclude BIMs that acquired phage resistance due to the activation of a CRISPR-Cas system, a colony PCR was performed with primers specific for three CRISPR loci in S. thermophilus (50). The PCRs were prepared using PCR master mix (Roche, Germany) with the following conditions: 94°C for 2 min, followed by 30 cycles of 94°C for 45 s, either 48°C (CR2 and CR3) or 51°C (CR1) for 45s, and 72°C for 2 min, with a final extension of 72°C for 5 min. The PCR products were visualized on a 1% tris-acetate-EDTA (TAE) agarose gel.

To perform full genome sequencing, DNA from the selected S. thermophilus strains was isolated by using the DNA DNeasy blood and tissue kit with the protocol for Gram-positive bacteria (Qiagen, Germany) and sent for sequencing on the Illumina MiSeq platform with 2 × 250-bp paired-end sequencing (Illumina, USA).

Sequencing reads were trimmed, analyzed, and assembled with the CLC Genomics Workbench 10.1.1 (Invitrogen). The assembled contigs were annotated by RASTtk (68). Single nucleotide polymorphism (SNP) analyses of WTs and BIMs were performed with CLC Genomics Workbench 10.1.1 (Invitrogen). The detected mutations were further evaluated to exclude false hits, i.e., SNPs at the ends of contigs, SNPs in noncoding regions not related to a putative promoter or a putative terminator, SNPs at mobile element proteins, and SNPs not resulting in amino acids changes. The analysis was performed by using CLC Main Workbench 7.7.3 (Invitrogen). The revised mutations were additionally verified by PCR, followed by Sanger sequencing (Macrogen, The Netherlands).

### Accession number(s).

The gene sequences reported in Table 3 together with the corresponding gene sequences in the WT strains are available in GenBank under the accession numbers MH700463 to MH700476.

## Supplementary Material

Supplemental file 1
