# Supplementary material for: Cell Wall Glycans Mediate Recognition of the Dairy Bacterium Streptococcus thermophilus by Bacteriophages
Source: Appl Environ Microbiol. 2018 Nov 15;84(23):e01847-18. doi: 10.1128/AEM.01847-18 (PMC6238053; doi:10.1128/AEM.01847-18)
Supplement: Supplemental file 1 [file zam023188871s1.pdf]

## Supplemental material

TABLE S1 Results of a volumetric viscosity test performed for stain STCH_09 and its phage resistant mutants (BIMs) to assess the difference in the exocellular polysaccharides (free EPS) production.

| **Strain** | **Viscosity time 1 [s]** | **Viscosity time 2 [s]** | **Viscosity time 3 [s]** | **Viscosity time average [s]** | **St. dev.** | **Viscosity** |
|---|---|---|---|---|---|---|
| STCH_09 | 42 | 43 | 42 | 42 | 1 | normal |
| STCH_09_BIM | 46 | 46 | 46 | 46 | 0 | enhanced |
| STCH_09_BIM_1 | 53 | 50 | 50 | 51 | 2 | enhanced |
| STCH_09_BIM_2 | 39 | 37 | 39 | 38 | 1 | normal |
| STCH_09_BIM_3 | 48 | 48 | 43 | 46 | 3 | enhanced |
| STCH_09_BIM_4 | 42 | 42 | 37 | 40 | 3 | normal |
| STCH_09_BIM_5 | 33 | 33 | 32 | 33 | 1 | reduced |
| STCH_09_BIM_6 | 44 | 42 | 38 | 41 | 3 | normal |

TABLE S2 List of mutations detected in phage resistant mutants (BIMs) generated in this study and confirmed by Sanger sequencing.

| **BIM** | **Mutated gene** | **Type of mutation**[a] |
|---|---|---|
| STCH_09_BIM | Glycosyltransferase *epsH* | AA substitution |
| | Glycosyltransferase *rgp* | Gene truncation |
| STCH_13_BIM | Tyrosine-kinase epsD | AA insertion |
| | Type I R-M system, DNA-methyltransferase subunit M | AA substitution |
| STCH_14_BIM | Glycosyltransferase *epsE* | Gene truncation |
| | O-antigen export system permease protein | AA substitution |
| | Two-component sensor kinase | AA substitution |
| STCH_15_BIM_1 | Glycosyltransferase *epsE* | Gene deletion |
| | 5'-nucleotidase family protein in cluster with NagD-like phosphatase | Gene truncation |
| | Zinc protease | AA substitution |
| | Ribose-phosphate pyrophosphokinase | AA substitution |
| STCH_15_BIM_2 | Glycosyltransferase *epsK* | AA substitution |
| | Guanylate kinase | AA substitution |
| STCH_15_BIM_3 | Glycosyltransferase *epsK* | Gene truncation |
| STCH_15_BIM_4 | Glycosyltransferase *epsK* | Gene truncation |
| STCH_15_BIM_5 | Glycosyltransferase *epsK* | Gene truncation |
| | Guanylate kinase | AA substitution |

[a]AA stands for amino acid

TABLE S3 List of samples collected during purification of cellular fractions with their relevant characteristics.

| Purification step no. | Sample name | Cell wall components present in the sample[a] |
|---|---|---|
| 1 | Cells in exponential phase | surface enzymes, membrane, membrane proteins, cell wall proteins, EPS/CPS, LTA, WTA, WPS, PG |
| 2 | Cells devoid of surface enzymes, membrane and membrane proteins | cell wall proteins, CPS, LTA, WTA, WPS, PG |
| 3 | Purified cell walls | CPS, WTA, WPS, PG |
| 4 | Purified peptidoglycan | PG |

[a]Cell wall components: exocellular polysaccharides (EPS/CPS), lipoteichoic acids (LTA), wall teichoic acids (WTA), polysaccharides intercalated with cell walls (WPS), peptidoglycan (PG)

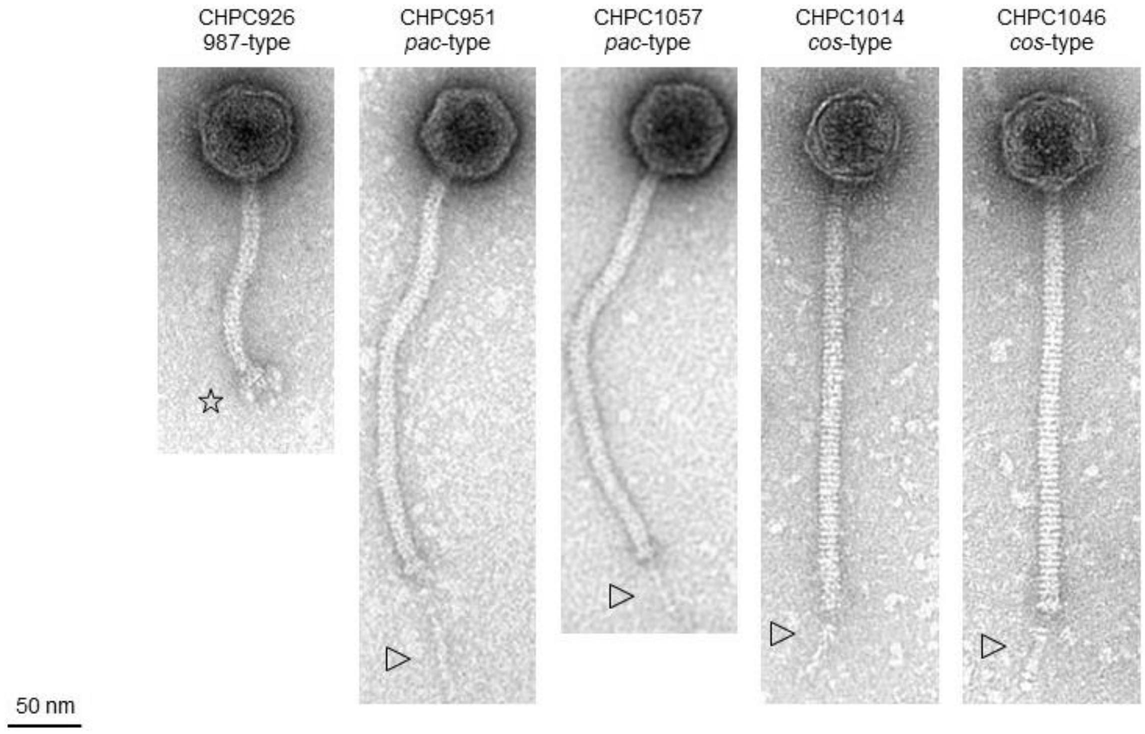


| | CHPC926 | CHPC951 | CHPC1057 | CHPC1014 | CHPC1046 |
|---|---|---|---|---|---|
| | n = 7 | n = 8 | n = 8 | n = 8 | n = 11 |
| Head diameter | 62 ± 1 | 59 ± 2 | 59 ± 2 | 58 ± 4 | 58 ± 2 |
| Tail length | 126 ± 3 | 252 ± 2 | 247 ± 2 | 241 ± 9 | 245 ± 4 |

**FIG S1 Transmission electron micrograph images of *S. thermophilus* phages used in this study.** The symbols indicate baseplate structures (asterisk) and a single central tail fiber (triangle). Head and tail sizes are indicated in nm. Images of CHPC926, CHPC1014, CHPC1046 were published previously (Szymczak *et al.* 2017).

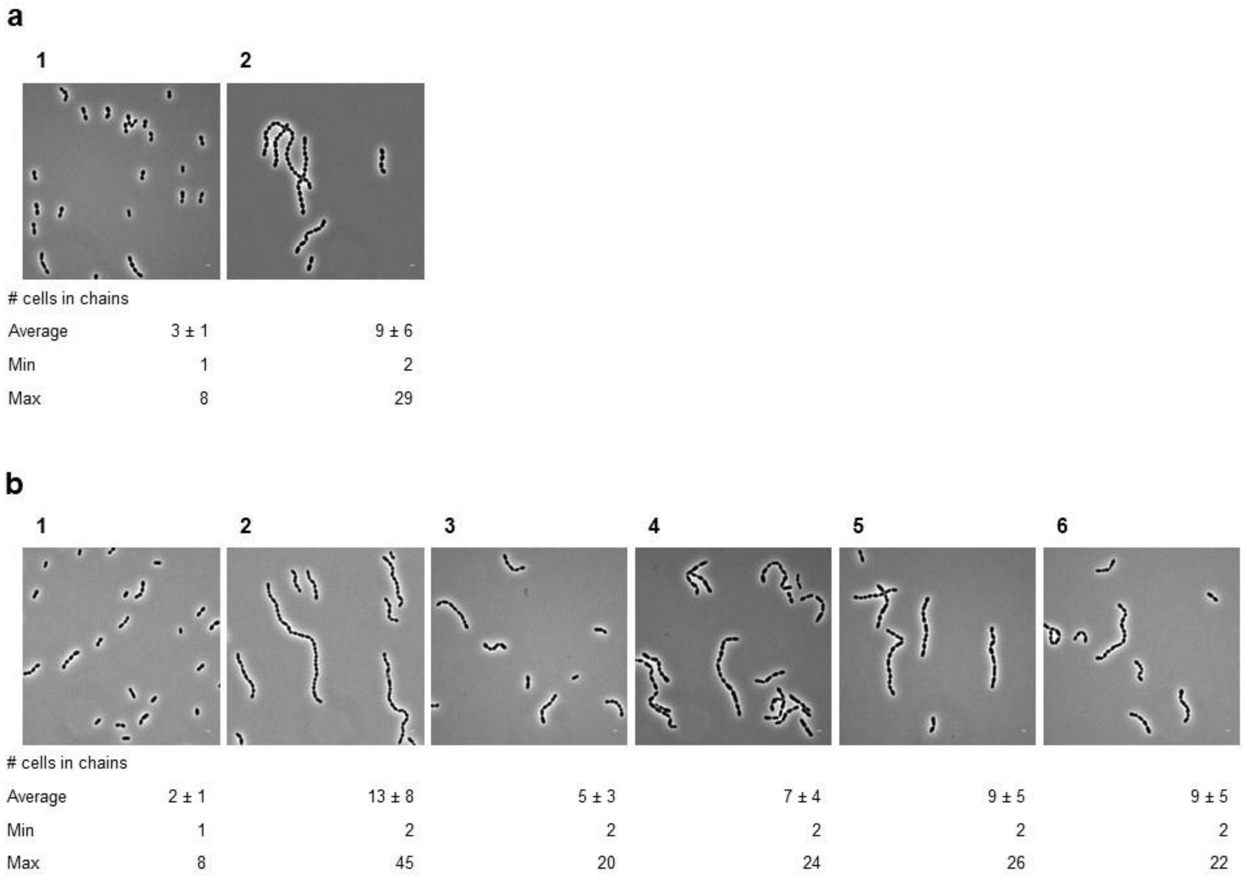


**FIG S2 Changes in chain lengths of *S. thermophilus* wild type strains and their phage-resistant mutants.** (a) STCH_14 forms short chains (1) and its phage-resistant mutant STCH_14_BIM forms elongated chains (2). (b) STCH_15 forms short chains (1) and its phage-resistant mutants STCH_15_BIM_1, STCH_15_BIM_2, STCH_15_BIM_3, STCH_15_BIM_4, STCH_15_BIM_5 form elongated chains (panels no. 2-6, respectively). For each strain, cells in 100 chains were counted to calculate the average number of cells per chain. Scale bars: 1 µm.

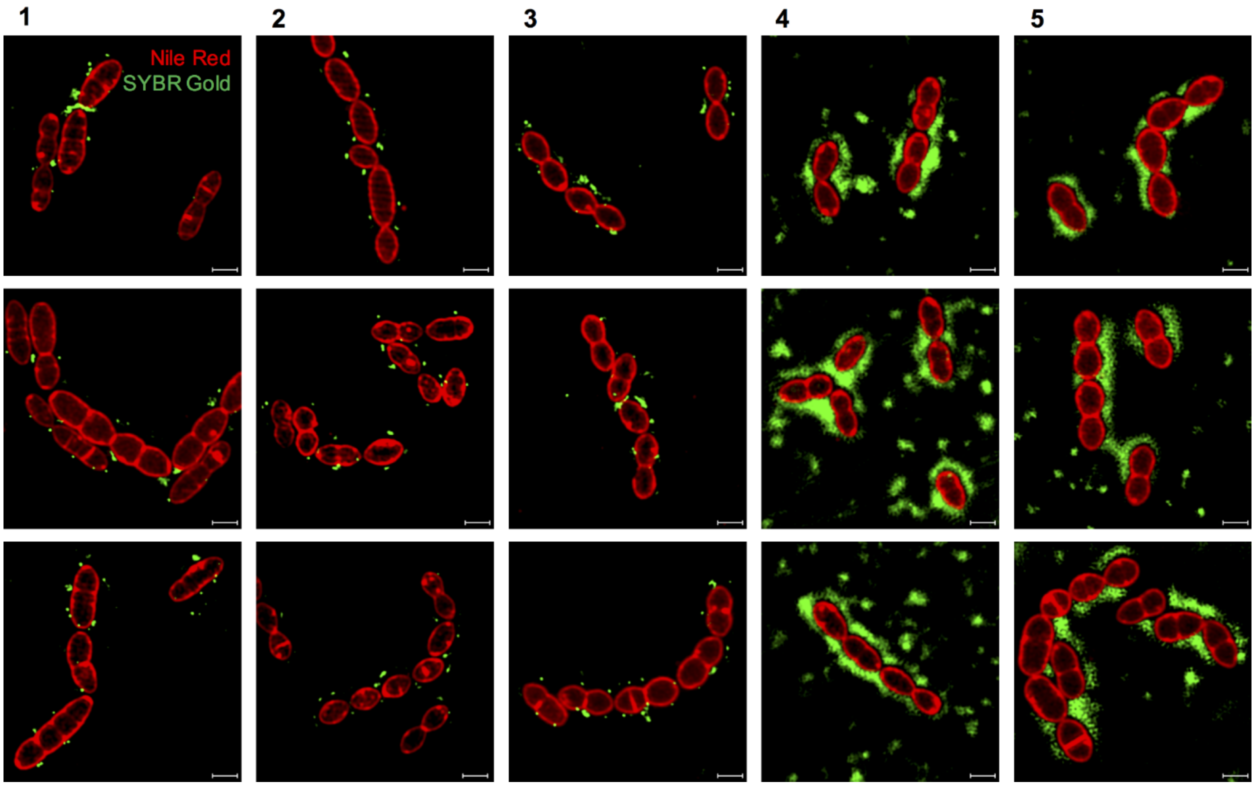


**FIG S3 Super-resolution structured illumination microscopy (SR-SIM) images of phage binding to *S. thermophilus* strains.** Bacterial cells were stained with Nile Red (red) and mixed with SYBR Gold DNA-labeled phages (green). Panels with phages and their host strains: (1) CHPC926 and STCH_15, (2) CHPC951 and STCH_12, (3) CHPC1057 and STCH_09, (4) CHPC1014 and STCH_13, (5) CHPC1046 and STCH_14. Two binding patterns are observed: spotty (panels no. 1, 2, 3) or diffused (panels no. 4, 5). Scale bars: 1 µm.

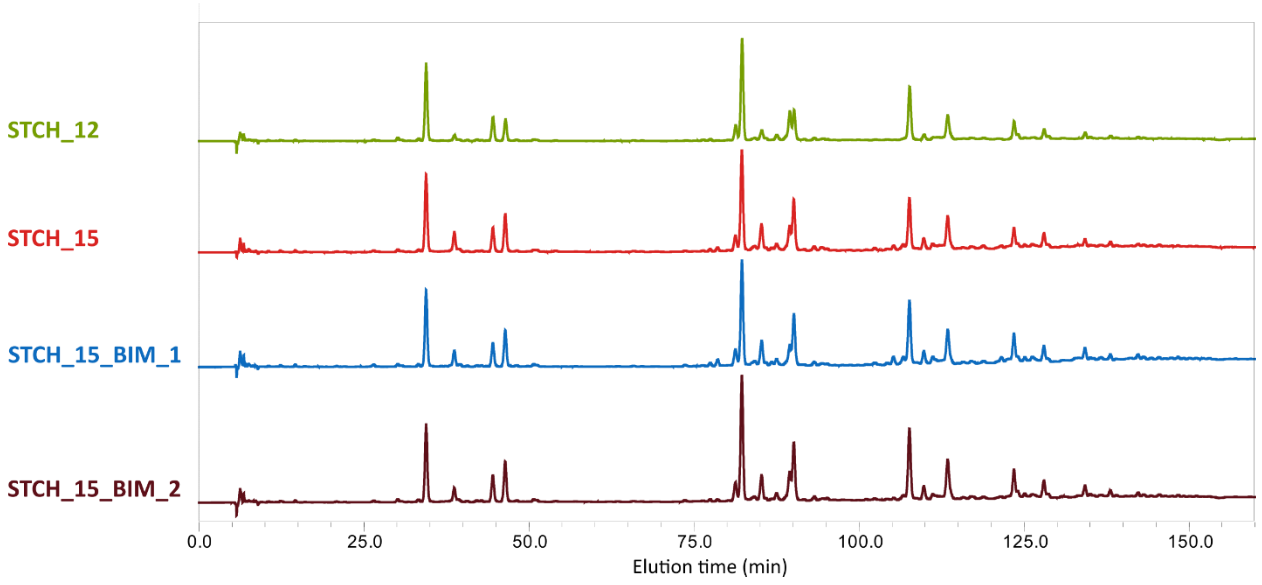


**FIG S4 HPLC profiles of muropeptides in peptidoglycan isolated from *S. thermophilus* strains.** Analysis was performed for two wild type strains (STCH_12 and STCH_15) and two phage-resistant mutants (STCH_15_BIM_1 and STCH_15_BIM_2).

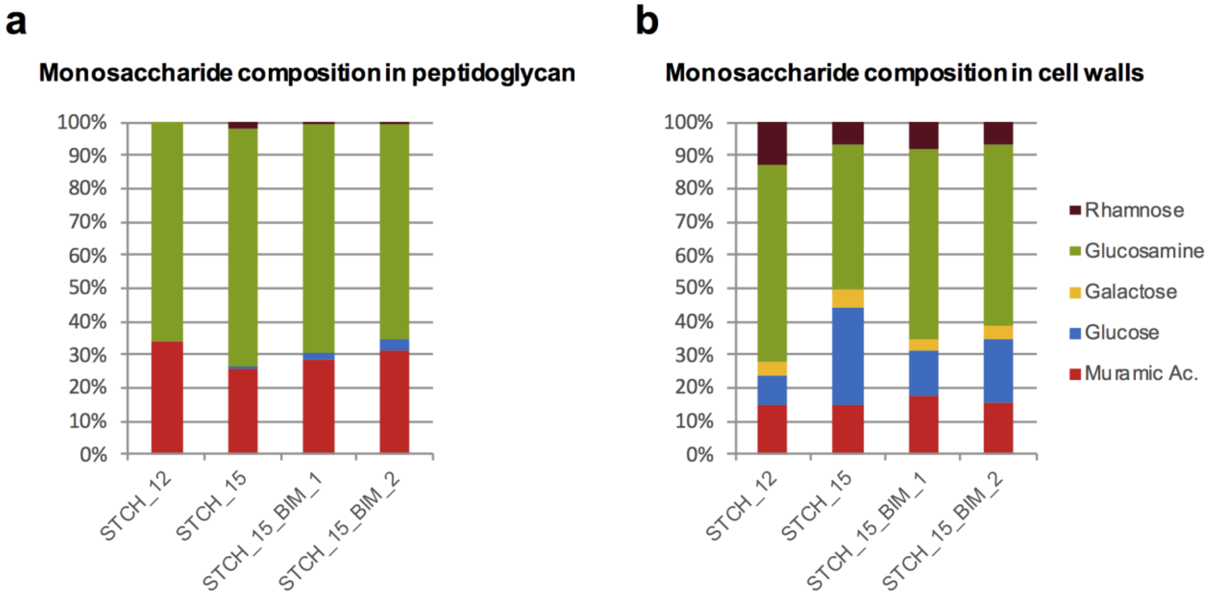


**FIG S5 Monosaccharide compositions in peptidoglycan and cell walls isolated from *S. thermophilus* strains.** Cellular fractions were purified from two wild type strains (STCH_12 and STCH_15) and two phage-resistant mutants (STCH_15_BIM_1 and STCH_15_BIM_2). Values were calculated based on individual peak areas compared to the total peak area from HPAEC-PAD analyzes preformed for: (a) purified peptidoglycan, (b) purified cell walls. Standards for different saccharides were eluted under the same conditions to allow peak identification.

## References

Szymczak P, Janzen T, Neves AR, Kot W, Hansen LH, Lametsch R, Neve H, Franz CMAP, Vogensen FK. 2017. Novel variants of *Streptococcus thermophilus* bacteriophages are indicative of genetic recombination among phages from different bacterial species. Appl Environ Microbiol 83:1–16.
